# Design, Development, and Optimization of Polymeric Based-Colonic Drug Delivery System of Naproxen

**DOI:** 10.1155/2013/654829

**Published:** 2013-10-01

**Authors:** Pooja Sharma, Anuj Chawla, Pravin Pawar

**Affiliations:** Chitkara College of Pharmacy, Chitkara University, Chandigarh-Patiala National Highway, Rajpura, Patiala, Punjab 140401, India

## Abstract

The aim of present investigation deals with the development of time-dependent and pH sensitive press-coated tablets for colon specific drug delivery of naproxen. The core tablets were prepared by wet granulation method then press coated with hydroxypropyl cellulose (HPC) or Eudragit RSPO : RLPO mixture and further coated with Eudragit S-100 by dip immerse method. The *in vitro* drug release study was conducted in different dissolution media such as pH 1.2, 6.8, and 7.4 with or without rat caecal content to simulate GIT conditions. Surface morphology and cross-sectional view of the tablets were visualized by scanning electron microscopy (SEM). All prepared batches were in compliance with the pharmacopoeial standards. The tablets which are compression coated with HPC followed by Eudragit S-100 coated showed highest *in vitro* drug release of 98.10% in presence of rat caecal content. The SEM of tablets suggested that the number of pores got increased in pH 7.4 medium followed by dissolution of coating layer. The tablets coat erosion study suggested that the lag time depends upon the coating concentrations of polymers. A time-dependent hydrophilic polymer and pH sensitive polymer based press-coated tablets of naproxen were promising delivery for colon targeting.

## 1. Introduction

Oral route of administration always receives more attention in comparison to the other drug delivery approaches [1]. Oral site-specific drug delivery systems to the colon have been gaining interests during the past two decades [2]. Colon specific drug delivery system offers several advantages in the treatment of colonic diseases such as ulcerative colitis, amoebiasis, Crohn's disease, irritable bowel syndrome, and colorectal cancer [3]. Delivery of drugs to the colon helps in reducing side effect thereby, achieving high local drug concentration at the afflicted site in the colon, hence resulting in optimal therapeutic effectiveness and good patient compliance [4, 5]. Different types of dosage forms have been used such as microspheres, nanoparticles, capsules, and hydrogels, have been used for colon specific drug delivery system [6–9]. However, recently more emphasis was laid on the microporous osmotic tablet, matrix tablets, and compression-coated tablets for colon specific drug delivery system because they are convenient to manufacture and produces greater flexibility in designing the dosage form rather than other novel drug delivery systems [10–12]. The various approaches that have been studied for colon drug delivery system (CDDS) via oral route include use of pH-sensitive polymers (methacrylic resins and cellulose acetate phthalate) [13, 14], time-dependent delivery which includes use of hydroxypropyl cellulose and hydroxypropyl methyl cellulose [15, 16], and pressure-dependent system [17]. 

 Time-dependent drug release systems can be formulated by applying coats onto drug cores which are capable of delaying the release through different mechanisms [18]. However, drawback associated with these deliveries is the lack of site specificity due to large variation in gastric emptying time. Thus, time controlled and site specificity are difficult. A simple pH-dependent approach is also not suitable to be used alone because of premature release of drug. Therefore, these problems can be overcome by using the combination of both time-dependent and pH-dependent polymers [19]. 

HPC is primarily used as a pharmaceutical additive for various purposes such as a tablet binder, film-coating material, and as a delayed release system [20]. The use of hydroxypropyl cellulose (HPC) has been reported earlier as a time released preparation, and the *in vivo* studies on beagle dogs demonstrated that the HPC press-coated tablets showed lag time of 4 h [21]. The molecular weight of HPC-EF has greater influence on its compactibility properties that is, the compactibility of HPC-EF increases due to its low molecular weight [22–24]. Therefore, taking into consideration all the properties, of HPC-EF, the core tablet of naproxen sodium was press coated with hydroxypropylcellulose which is a pH-independent hydrophilic and time-dependent polymer and releases the drug after predetermined lag time. 

 However, the lag time of 4 h was not sufficient to target the drug delivery to colon. Therefore, the press-coated tablets were coated with Eudragit S-100 coating which retarded the release in upper part of GIT and showed the lag phase of 6 h, assuring sufficient lag time for delivering the drug to the colon [25]. 

 Taking the above information in view, the present investigation based on the utilization of time-dependent polymer such as hydroxypropyl cellulose and pH-sensitive polymer, that is, Eudragit S-100 for colon drug delivery of naproxen sodium. 

 Naproxen sodium, 2-napthaleneacetic acid, and 6 methoxy-a-methyl sodium salt are a nonselective COX inhibitor widely used as analgesic and in treatment of rheumatoid arthritis, colitis [26]. Moreover, because of the same mode of action, it shows synergistic action with that of anticancer drugs [27]. Piao et al. prepared a coated naproxen tablets to reduce intestinal tissue damage by delivering the drug specifically to colon for the treatment of colonic diseases like colitis and crohn's disease [28]. 

 Therefore, by considering all the above data, the naproxen sodium was used as a model drug in colon delivery system using two different polymers: HPC-EF and Eudragit S-100, so that it can deliver the drug locally in colon. Such type of formulation also minimizes upper GI tract side effects of naproxen sodium and help in providing therapeutic benefits.

## 2. Materials and Methods

### 2.1. Materials

Naproxen sodium was obtained as a complimentary sample by Microlabs Pvt Ltd., Banglore, India. Hydroxypropylcellulose EF (HPC) was received from Matrix Lab, Hyderabad, India. Eudragit S-100, Eudragit RLPO, and Eudragit RSPO were procured from Evonik industries, Mumbai, India. All other chemicals used were of analytical grade.

### 2.2. Methods

#### 2.2.1. Preparation of Naproxen Sodium Core Tablets

The core tablets of naproxen sodium were prepared by wet granulation. The weighed quantity of drug and lactose was mixed uniformly and granulated using starch paste as binding agent. The starch paste (5%) was prepared by dissolving corn starch powder in required quantity of warm distilled water. The wet mass was passed through 22# sieve, and the granules were dried in a tray drier for 15 min at 45°C. The dried granules were then passed through 22# sieve and mixed uniformly with magnesium stearate as lubricant. The prepared granules were then compressed on multiple punch tablet machine (AK Industries, Nacodar, India) using 8 mm concave punches.

#### 2.2.2. Preparation of Press-Coated Core Tablets

The core tablets were press coated with a polymers hydroxypropylcellulose/Eudragit RSPO : RLPO (Table 1). One half of the polymer was filled into the die cavity to make a powder bed at bottom of a single punch tablet machine using a flat punch of 12 mm diameter. The core tablet was placed in the centre on the above powder bed, followed by filling of the remaining half quantity of the polymer. The tablet was then compressed and the press-coated tablets were evaluated and used for further coating.

#### 2.2.3. Enteric Coating of Press-Coated Tablets

The press-coated tablets were further coated with pH-dependent polymer Eudragit S-100 using dip immerse method. A different concentration (i.e., 2.5, 5.0, and 7.5%) of coating solution of Eudragit S-100 was prepared in a mixture of isopropyl alcohol (IPA) and acetone. The coated tablets were dried at room temperature for 24 hrs and kept in vacuum dessicator.

#### 2.2.4. Evaluation of Granules

*Angle of Repose. *Angle of repose is defined as the maximum angle possible between the surface of pile of powder (5 gm) and horizontal plane. The angle of repose is used to determine the flow characteristics of the powder. This can be calculated by the following formula:

(1) $$\begin{array}{c} \tan\theta =\frac{h}{r}, \end{array}$$

where *θ* is angle of repose, *h* is height of pile, and *r* is radius of pile.

*Bulk Density. *A weighed amount (9–13 gm) of granules was poured in the graduated measuring cylinder (25 mL capacity). The initial volume of poured granules was then noted. Calculate the bulk density using the following formula:

(2) Bulk  density  (BD)=Mass  of  granulesInitial  volume  of  granules.

*Tapped Density. *A weighed amount (9–13 gm) of granules was poured in measuring cylinder (25 mL capacity). The cylinder is then mechanically tapped after observing the initial volume. Noted the volume or mass readings of tapped granules until little further volume or mass change is observed:

(3) Tapped  density=Mass  of  granulesTapped  volume  of  granules.

*Compressibility Index. *The compressibility index of granules can be determined by Carr's compressibility index by the equation:

(4) Carr's index  (%)=  (Vo−Vf)Vo×100,

where *V*_*o*_ is unsettled apparent volume and *V*_*f*_ is final tapped volume.

*Hausner's Ratio. *It was determined by the ratio of tapped density to bulk density:

(5) Hausner's ratio=Tapped  densityBulk  density.

#### 2.2.5. Drug Excipient Compatibility Study

*Fourier Transform Infrared Spectroscopy (FTIR). *The infrared spectra of naproxen sodium, hydroxypropyl cellulose, Eudragit S-100, physical mixture of press-coated tablet (naproxen sodium, hydroxypropyl cellulose), and physical mixtures of coated tablet (naproxen sodium, hydroxypropyl cellulose, and Eudragit S-100) were recorded in the range of 4000 to 400 cm^−1^ using FTIR spectrophotometer (Mode spectrum RX 1, Perkin Elmer, UK). The IR spectra for the samples were obtained by potassium bromide (KBr) disk method.

#### 2.2.6. Evaluation of Tablets

*Weight Variation. *Twenty tablets from each formulation were randomly selected and individually weighed. The average weight and standard deviation was also calculated. 

*Hardness. *The tablet crushing strength was determined by Monsanto hardness tester (Interlabs, Ambala, India). A tablet is placed between the anvils, and reading was noted of the force which causes tablet to break.

*Tablet Dimensions. *The diameter and thickness of the tablets were determined using a digital vernier calliper (CD-6′′CSX, Mitutoyo Digimatic Calliper, Japan).

*Friability. *Six tablets from each batch were weighed and placed in Roche friabilatar (902, EI Products, Panchkula, India). The apparatus was rotated at 25 rpm for 4 min. After rotations the tablets were dedusted and again weighed. The percentage friability of the tablets was measured by using the following formula:

(6) %  Friability=Initial  weight−Final  weight  Initial  weight×100.

#### 2.2.7. Determination of Drug Content

Twenty tablets from each formulation were weighed and finely powdered using mortar and pestle. A quantity equivalent to 50 mg of naproxen sodium was transferred to 100 mL volumetric flask containing phosphate buffer pH 7.4 and mixed thoroughly. The solution was filtered and analyzed for its drug content using UV/Vis spectrophotometer (AU-2701, Systronics, Mumbai, India) at 272 nm.

#### 2.2.8. *In Vitro* Drug Release Studies

An *in vitro* drug release study was carried out using USP type II dissolution apparatus (DS8000, LABINDIA, Navi Mumbai, India) in a 900 mL of dissolution medium at temperature 37 ± 0.5°C and stirring speed of 100 rpm. The dissolution test was performed in three different dissolution mediums, that is, pH 1.2, 6.8, and 7.4, in order to mimic the GIT conditions. The tablets were first kept in pH 1.2 (0.1 N HCl) for 2 h. After 2 h the dissolution media were replaced with phosphate buffer pH6.8, and dissolution was carried out for 3 h; then the pH of dissolution media was adjusted to 7.4, and the drug release study was continued up to 24 h. A 5 mL of sample was withdrawn from the dissolution media at a specified time intervals, followed by replacing with same aliquot of fresh dissolution media to maintain sink conditions. The samples were then analysed using UV/Vis spectrophotometer at 272 nm. The *in vitro* dissolution study was performed in triplicate [29].

*Preparation of Rat Caecal Content Medium. *The rat caecal content medium has the similar contents to that of the intestinal microflora; therefore it was prepared to study further drug release of tablet and to assess the susceptibility of formulation to colonic bacteria. Wistar rats weighing 100–150 g, maintained on normal diet and 1 mL of 1% w/v solution of HPC/Eudragit S-100 in water, were administered with the help of Teflon tubing directly into the stomach region via oral cavity. The treatment was continued for 6 days to induce enzyme responsible for degradation of HPC/Eudragit S-100. At least 45 min before the commencement of dissolution study, the rats were sacrificed, and the abdomen was cut to isolate caecum. The caecal content was immediately transferred into a tared beaker containing phosphate buffer pH 7.4 previously bubbled with nitrogen to obtain 4% w/v concentration caecal content equivalent to 8 g that was added to 200 mL of buffer to give a final caecal dilution of 4% [30].

#### 2.2.9. Drug Release Study in Presence of Rat Caecal Content

The drug release study in presence of caecal content was performed on batches NF2 to NF5 using dissolution test apparatus with slight modifications in a procedure. The initial studies were carried out in same manner, that is, in pH 1.2 for 2 h and pH 6.8 for 3 h. After 5 h tablets were placed in 200 mL phosphate buffer pH 7.4 containing rat caecal medium, and release studies were carried out till 24 h. The required amount of samples (2 mL) was taken at specified time intervals and replenished with same volume of fresh dissolution media maintained at 37 ± 0.5°C. The experiment was carried out in the presence of continuous supply of nitrogen. The samples were then analyzed using a UV spectrophotometer [31].

#### 2.2.10. Scanning Electron Microscopy

The tablets of optimized batch were removed from the dissolution apparatus at predetermined time intervals, and their surface morphology and cross-sectional view were then visualized using scanning electron microscope (JSM 6100 Jeol, Japan). The gold sputter coating of samples was done prior to examination to make them electrically conductive.

#### 2.2.11. Coat Erosion Study

The coat erosion study was performed in the same manner as the dissolution study at pH 6.8 and pH 7.4 phosphate buffer media [32]. Before proceeding dissolution study, the initial weight of the tablet was measured (*W*_*i*_). At a specified time interval, tablets were taken out from the media, dried at 60°C (until constant weight was not achieved), and reweighed (*W*_*d*_). The % coat erosion was calculated using the following formula [33]:

(7) Coat  erosion  (%)=WdWi×100.

#### 2.2.12. Drug Release Kinetics

To study the drug release kinetics, the *in vitro* release data were fitted to zero order (cumulative percentage of drug released versus time), first order (log cumulative percent of drug remaining to be released versus log time), and Higuchi kinetics (cumulative percent of drug release versus square root of time).

 The drug release mechanism was determined using Korsmeyer-Peppas equation by plotting the graph between log percentage of drug released versus log time. The exponent “*n*” indicates the mechanism of drug release calculated through the slope of the straight line:

(8) $$\begin{array}{c} \frac{M_{t}}{M_{f}}-Kt^{n}, \end{array}$$

where *M*_*t*_ is amount of drug release at time *t*,  *M*_*f*_ is amount of drug release at infinite time,  *K* = release rate constant,  and *n* is diffusional exponent indicates the mechanism of drug release.

 If value of *n* falls between 0.5 and 1.0; it is termed as non-fickian release, while in case of Fickian diffusion, *n* = 0.5. For zero order release case II transport *n* = 1; on the other hand if *n* > 1 then it indicates supercase II transport [34].

#### 2.2.13. Stability Studies

The stability study was conducted according to ICH guidelines. All the formulations were stored in aluminium packaging laminated with polyethylene and kept in humidity chamber at accelerated and room temperature conditions for 6 months. The samples were withdrawn at a specified time intervals of 0 days, 1 month, 2 month, 3 month, and 6 month. The samples were evaluated for their physical characteristics (colour) and drug content. The degradation rate constant (*K*_cal_), shelf life (*t*_90_), and initial drug concentrations providing 2 years shelf life (Int_cal_) were determined [35]. 

## 3. Results

The naproxen sodium is a nonsteroidal anti-inflammatory drug and has poor compressibility. Therefore, the granules of naproxen sodium were prepared by using diluents and binding agent. The granules used for preparing the core tablets of naproxen sodium were evaluated for angle of repose, bulk density, tapped density, compressibility index, and Hausner's ratio. The angle of repose was found to be 23.41° ± 1.32 exhibited the excellent flow properties. The packing properties of the material can be evaluated by the bulk and tapped density. The bulk density and tapped density were 0.36 ± 0.004 gm/mL, and 0.40 ± 0.003 gm/mL, respectively, which indicate good flowability of the granules [36]. Moreover, the compressibility index also known as Carr's index was found to be 9.84 ± 1.09, and Hausner's ratio (1.15 ± 0.03) also given in Table 2 indicates that the granules formed were easily compressible.

### 3.1. Fourier Transform Infrared Spectroscopy (FTIR)

**Figure 1:**
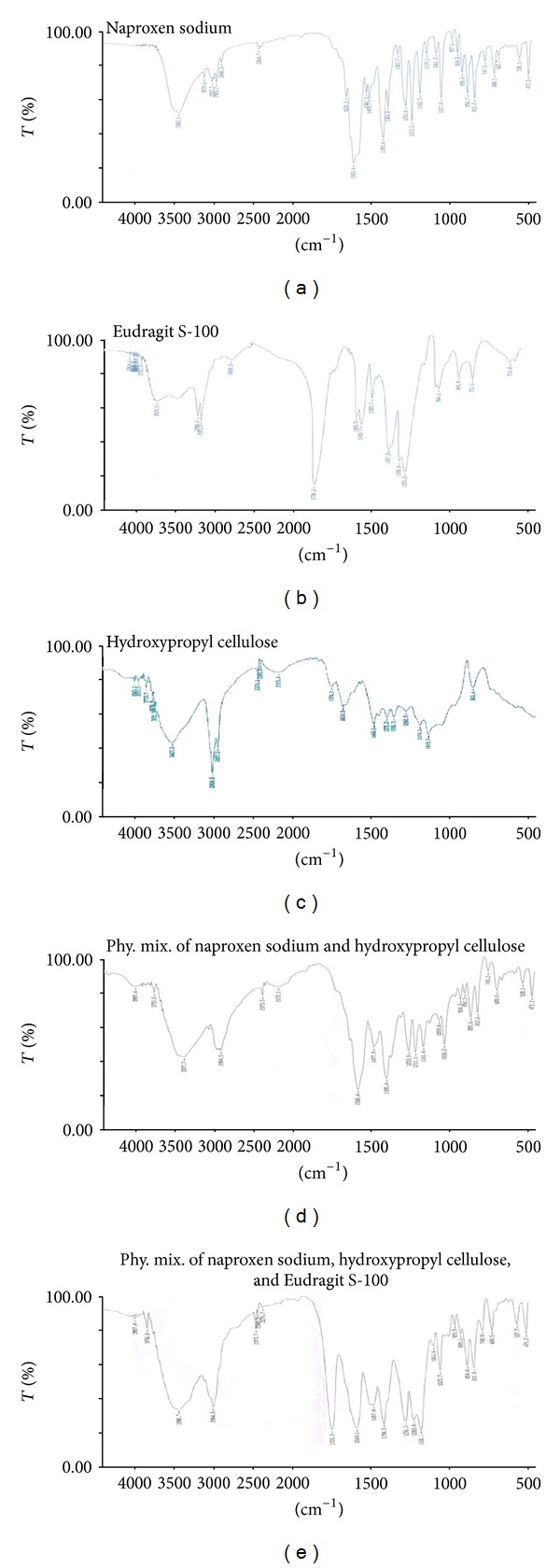
Fourier transform infrared spectra of naproxen sodium, Eudragit S-100, hydroxypropyl cellulose, physical mixture (naproxen sodium and hydroxyl propyl cellulose), and physical mixture (naproxen sodium, hydroxyl propyl cellulose, and Eudragit S-100).

FT-IR spectra of the drug, polymers, and their physical mixtures are depicted in Figure 1. The drug sample showed characteristic functional group peaks at 1252 cm^−1^ due to C–O stretching (acid), 1583 cm^−1^ due to COO– stretching, C–C aromatic stretching at 1631 cm^−1^, and C–H aliphatic stretch at 2840 cm^−1^. HPC shows a broad peak at 3460 cm^−1^ which may be due to O–H stretching vibration, whereas the peak shown at 2924 cm^−1^ is present due to C–H asymmetric stretching vibration. The peaks appeared at 1770 cm^−1^, and 1260 cm^−1^ was observed due to C=O stretching and C–O stretching, respectively. In physical mixture of press-coated tablet, the peaks at 1253.9 cm^−1^, 1580.4 cm^−1^, 855.6 cm^−1^, and 2924.8 cm^−1^ indicate the presence of naproxen sodium and hydroxypropyl cellulose without any ineraction. The FT-IR of physical mixture of coated tablets showed the characteristic peak of the drug, and polymers revealed that all peaks were easily detectable in the physical mixture. This shows that there is no interaction between drug and excipient.

### 3.2. Physicochemical Properties of Naproxen Sodium Tablets

The physicochemical properties of prepared naproxen sodium press-coated tablets were depicted in Table 3. The weight variation values for all formulations (NF1 to NF9) were found between 251.24 and 508.15 mg. All the weight variation values of tablets were complying with official compendia, and there were no effect of press coating as well as Eudragit S-100 coating on tablet weight variation.

 The mean thickness of all the formulated batches of tablets was measured, and the results revealed that batch NF1 showed less thickness of 4.01 ± 0.02 mm, whereas the thickness of press-coated formulations NF2 and NF6 got increased, that is, 4.62 ± 0.02, and 4.57 ± 0.02 respectively. The thickness of press-coating tablets further increased, and the value varies from 4.63 ± 0.02 to 4.86 ± 0.02 mm. The diameter and friability of the batches were between 8.01 to 12.94 mm and 0.22 to 0.63%, respectively. On the other hand, the percentage friability that was less than 1% indicates that the friability is within the specified limit. The hardness of the core tablet usually kept low, that is, 4.09 ± 0.59, so that press-coated polymer HPC get sticks to the core tablet [37], whereas the hardness of the coated tablets varies from 6.05 to 6.59 kg/cm^2^. The formulation NF2 showed maximum % drug content, that is, 96.37 ± 1.91 as compared with other formulations of tablets. The remaining formulations showed the % drug content in-between the range of 94.36 ± 2.03 to 95.61 ± 1.96%.

### 3.3. *In Vitro* Drug Release Studies without Rat Caecal Content

**Figure 2:**
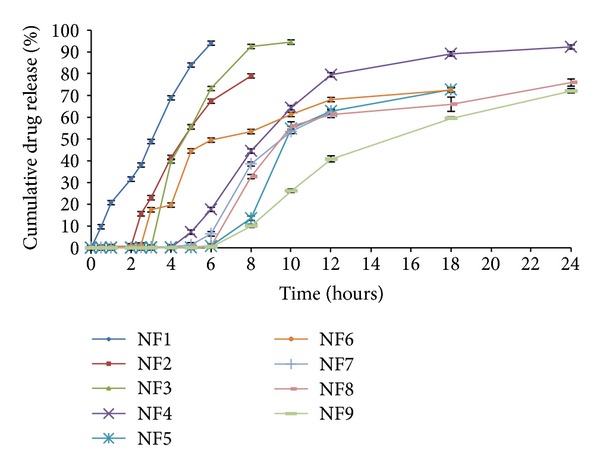
*In vitro* release profiles showing the cumulative percent of naproxen sodium release from core (NF1), press-coated tablets (NF2, NF6), and Eudragit S-100 coated tablets (NF3, NF4, NF5, NF7, NF8, and NF9) without rat caecal content. Data are expressed as mean ± S.D. (*n* = 3).

**Figure 3:**
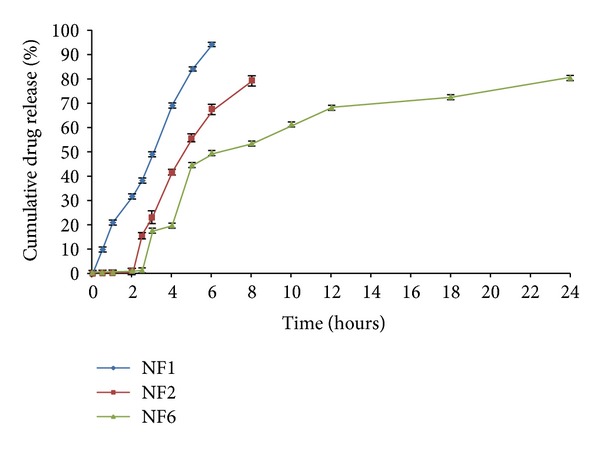
Comparative *in vitro* release profiles of naproxen sodium core tablets (NF1), press-coated HPC tablets (NF2), and press-coated Eudragit RSPO : RLPO tablets (NF6). Data are expressed as mean ± S.D. (*n* = 3).

**Figure 4:**
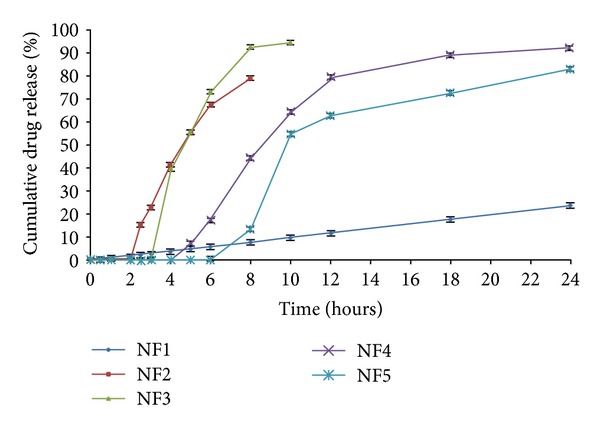
*In vitro* release profiles of naproxen sodium core tablets (NF1), press-coated HPC tablets (NF2), and press-coated HPC tablets enteric coated with Eudragit S-100 in different concentrations of 2.5%, 5.0%, and 7.5% (NF3, NF4, and NF5), respectively. Data are expressed as mean ± S.D. (*n* = 3).

**Figure 5:**
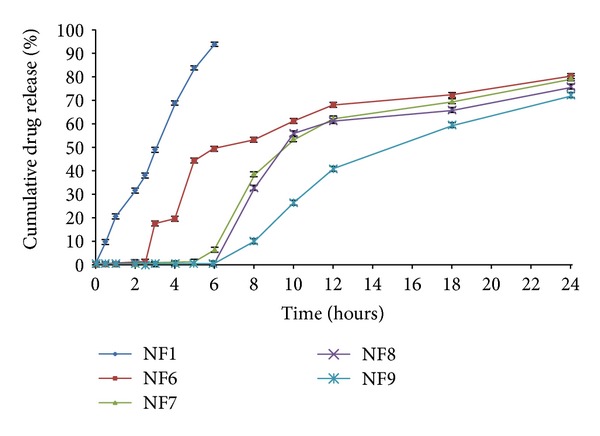
Comparison of *in vitro* release profiles of naproxen sodium core tablets (NF1), press-coated Eudragit RSPO : RLPO formulation (NF6), and press-coated Eudragit RSPO : RLPO tablets enteric coated with Eudragit S-100 in different concentrations of 2.5%, 5.0%, and 7.5% (NF7, NF8, and NF9), respectively. Data are expressed as mean ± S.D. (*n* = 3).

 The dissolution profile of core tablet (NF1) was studied in phosphate buffer pH 7.4 to examine the release profile of the core tablet. The study resulted in 94.05 ± 1.46% of drug release within a period of 6 h in pH 7.4 media (Figure 2). The press-coated tablets batch NF2 and NF6 were subject to dissolution studies at different pH media, that is, acid buffer media of pH 1.2, phosphate buffer pH 6.8, and pH 7.4. It was observed that NF2 formulation which was press-coated with HPC showed 79.01 ± 2.02% drug release for a period of 8 h. On the other hand, Eudragit RSPO : RLPO mixture press-coated batch NF6 exhibited 80.47 ± 1.11% of cumulative drug release for 24 h (Figure 3). The press coated tablets were enteric coated with different concentrations of Eudragit S-100 polymer given in Table 1. On analyzing % cumulative drug release, it was found that the formulations NF3, NF4, and NF5 demonstrated more drug release, that is, 94.54 ± 1.75, 92.32 ± 2.27, 83.19 ± 1.33%, respectively (Figure 4). Formulations NF7 to NF9 provide cumulative release in the range of 72.17 ± 1.02 to 79.47 ± 2.38 (Figure 5). However, these results suggest that the formulations NF4, NF5, NF7, NF8, and NF9 prevent the drug release in upper part of GIT, whereas NF3 started eroding in small intestine, that is, in pH 6.8.

### 3.4. *In Vitro* Drug Release Studies in the Presence of Rat Caecal Content

**Figure 6:**
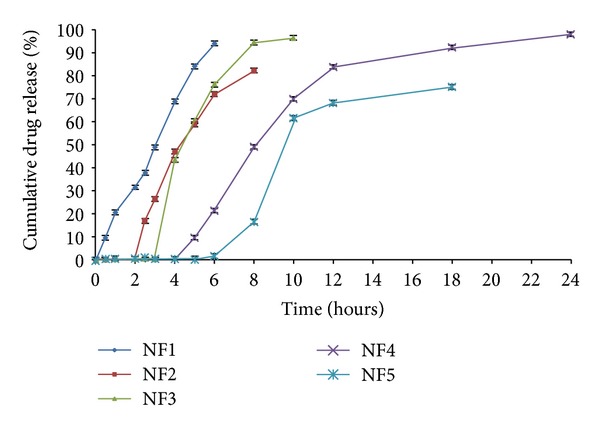
*In vitro* release profiles of various formulations of naproxen sodium (NF1, NF2, NF3, NF4, and NF5) in presence of 4% w/v rat caecal content. Data are expressed as mean ± S.D. (*n* = 3).

Eventually, the release of drug from formulations NF1 to NF5 was analyzed in colonic environment containing phosphate buffer pH 7.4 in the presence of 4% w/v rat caecal contents to precisely examine the release behaviour of these formulations in colonic environment. Amongst the formulations, the optimized formulation, that is, NF4, showed the maximum percentage of cumulative drug release 98.10 ± 1.61% in the colon region.  Figure 6 demonstrated that all the formulations of tablet showed more than 5% of drug release in the presence of rat caecal content in comparison to the *in vitro* drug release in the absence of rat caecal content which has been shown in Figure 2.

### 3.5. Scanning Electron Microscopy

**Figure 7:**
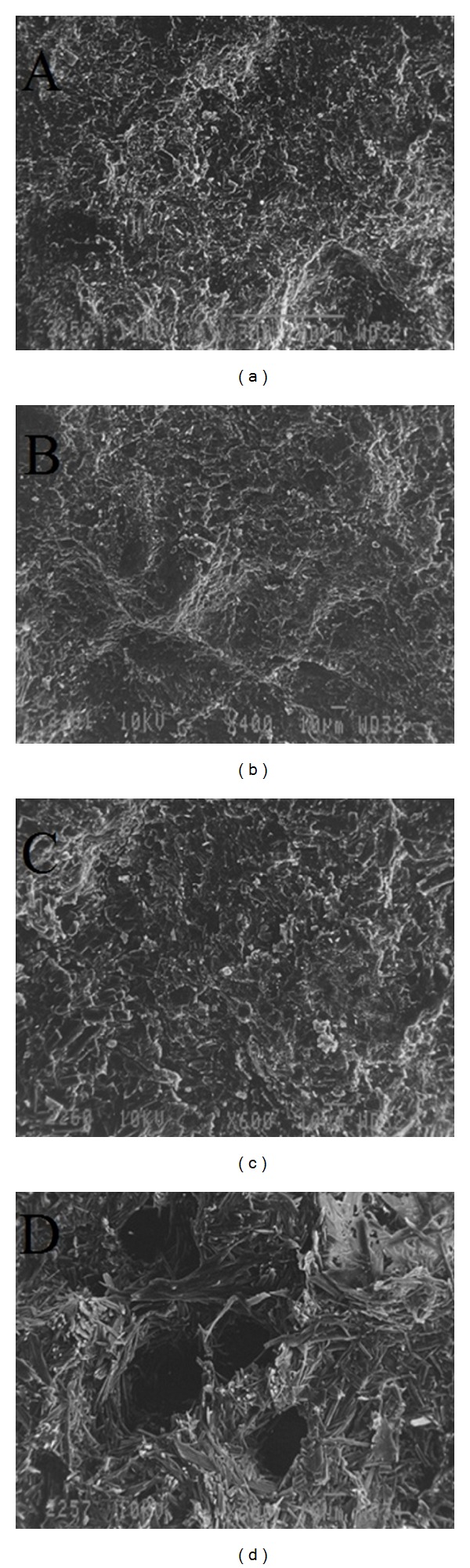
Scanning electron microscopy of optimized batch (NF4), obtained before dissolution (a), after 2 h dissolution at pH 1.2 (b), after 5 h dissolution at pH 6.8 (c), and during dissolution study at pH 7.4 (d).

The information regarding the different behaviour of the coated tablets was obtained by the SEM analyses. No pores were detected in the tablet before dissolution study. During dissolution studies, the SEM analyses of tablets were done at different type intervals and in different media conditions.  Figure 7 presented that the tablet does not erode in acidic media. However, very minute pores were seen in pH 6.8 which got increased in pH 7.4 phosphate buffer.

### 3.6. Coat Erosion Study

**Figure 8:**
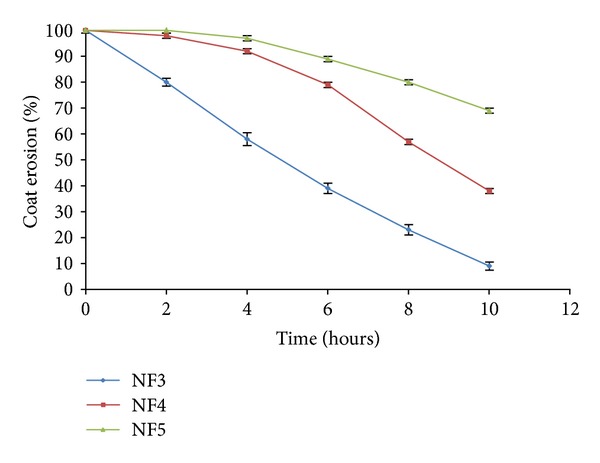
Coat erosion study of press-coated HPC tablets enteric coated with Eudragit S-100 in different concentrations of 2.5%, 5.0%, 7.5% (NF3, NF4, and NF5), respectively, without presence of rat caecal content. Data are expressed as mean ± S.D. (*n* = 3).

The coat erosion study was done in pH 6.8 and pH 7.4 for formulations NF3, NF4, and NF5 as shown in Figure 8. It was demonstrated that initially the coating remains 100% in all said three formulations. At pH 6.8, the behaviour of coating remains same for all formulations of tablet except NF3 batch. In case of NF3, the coating erodes fastly results only 41.0 ± 2.0% of coating was present on the surface of tablet at pH 7.4 as compare to other formulations. On the other hand in formulations NF4 and NF5, the erosion was observed in coating of negligible manner at pH 7.4 at initial stage. Later on at the time points 7 h and 9 h, the coating was decreased to half in case of NF4 and NF5 formulations, respectively.

### 3.7. Drug Release Kinetics

To determine the quantitative analysis of the values obtained from drug release profile, various mathematical models are used. The goodness of fit was evaluating using regression coefficient (*r* ^2^) values. The regression coefficients (*r* ^2^) for all the formulations using different kinetics equation are listed in Table 4. The table data revealed that *in vitro *release from the tablets is better explained by the Higuchi equation, where the rate constants obtained from the slope provide the highest linearity. To explore the drug release mechanism, *in vitro* release results were further fitted to the K-P model. This model analyses the release of polymeric dosage forms, either in cases when the release mechanism is not well known or when more than one type of release phenomena are involved. Among all formulations, the formulation NF1, NF2, and NF6 followed Fickian kinetics (*n* ≤ 0.45), and the remaining formulations followed non-Fickian kinetics (*n* ≥ 0.45).

### 3.8. Stability Studies

**Figure 9:**
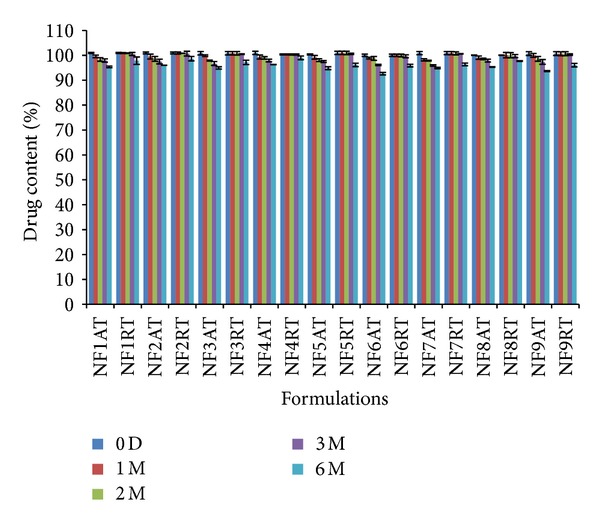
Stability of naproxen sodium tablets under accelerated condition and room temperature. Mean ± S.D. (*n* = 3). AT: accelerated temperature (40°C),  RT: room temperature,  D: days,  and M: months.

The stability studies of all the formulations were conducted at accelerated and room temperature storage conditions, and the tablets were examined for physical appearance and drug content. The results indicated that no change in physical appearance was noticed upon visual inspection of the tablets. All the formulations showed more than 90% of drug content during both accelerated and room temperature storage conditions (Figure 9). The degradation rate constants (*k*_cal_) and shelf life (*t*_90_) at room temperature for all formulations range between 1.11 to 2.60 day^−1^ and 403.84 to 945.94 days, respectively. 

## 4. Discussion

The micrometric properties of the prepared granules were found to be in the acceptable range. Angle of repose that was less than 25° indicates that flow behaviour of thegranules was found to be good [38]. The FTIR studies were done to find out the compatibility between drug excipients. From the results it was suggested that there was no interaction found between drug and polymer since the peaks of the drug still could be detected in the mixture.

 The weight variation and friability of all the formulations were less than 4%, and 0.4% respectively. The mean thickness of NF1 was less than other formulated batches due to the reason that batch NF1 consists of a core tablet; that is, no coating was applied over it due to its thickness which was least. The thickness value of batch NF2 and NF6 was increased due to press coating with hydroxypropyl cellulose and Eudragit RLPO and RSPO mixture, and further the remaining formulations (NF3, NF4, NF5, NF7, NF8, and NF9) exhibited coating of an enteric-coated polymer over the press-coated tablets which also results in increased thickness. This might be attributed to the fact that as the coating over the tablet increases, the thickness also gets increased that can affect release of drug from coated tablets in colonic media. It was observed that the diameter of the coated tablets was more compared to the core tablets due to the difference in their die cavities of the tablet punching machine and also due to Eudragit S-100 coating [39]. On the other hand, the percentage friability that was less than 1% indicates that the friability is within the specified limit. The starch paste concentration resulted in a significant effect on the hardness of the tablets [40]. The increase in concentration of starch paste which acts as a binder for preparing a granules results in more hardness of the tablets. The coating is another parameter which might be increase in hardness of the coated formulation. The content uniformity for all uncoated and coated tablets was observed with minimum variation and optimum range. The maximum drug content was obtained in formulation NF2, due to the press coating of hydrophilic polymer, that is, hydroxypropyl cellulose which resulted in more marked increase in drug release rate in phosphate buffer medium. 

The *in vitro* drug release studies were carried out of all the formulations in pH 1.2, 6.8, and 7.4, and it was observed that the tablets which were press coated with HPC or Eudragit RSPO : RLPO mixture delayed the drug release and prevent its release in gastric pH in comparison to the core tablet. However, due to more hydrophilic property of HPC than Eudragit RSPO : RLPO mixture, it showed high cumulative release (79.01 ± 2.02%) but does not remain in intact form in small intestine (pH 6.8). The tablets which were press coated with Eudragit RSPO : RLPO mixture also showed premature drug release followed by sustained action (Figure 3) for 24 h. Therefore, it was concluded that the coating of Eudragit S-100 was required to impart an enteric effect.

Figure 4 describes that the dissolution profile of formulations NF1 to NF5 revealed that the lag time of drug release increases with increasing the coating level of Eudragit S-100. At a coat concentration of 2.5% w/v (NF3), the lag time of 4 h was achieved; however, this lag time was insufficient to reach intact to the colon. Thus, 5% w/v and 7.5% w/v coating concentrations were needed to be applied on the press-coated tablets to prevent the drug release in upper part of GIT. By the 5% w/v coating (NF4), the tablet provides a desired lag time of 6 h and retarded the drug release in pH below 7.4 with cumulative drug release of 92.32 ± 2.27%. However, in case of 7.5% w/v coat level (NF5), the lag time was obtained up to 8 h with lesser cumulative release of 83.19 ± 1.33% in comparison to formulation NF4. This can be explained by the fact that on increasing the coat concentration, the coat becomes more impermeable thereby retarding the drug release. Thus, from above results it was concluded that the formulation NF4 maintained the integrity of the coats and prevented the drug release for upto 6 h. Finally, it was evident from the results that the tablet containing two polymeric systems showed much more site specificity than the single polymeric systems [41]. 

 The dissolution profile of formulations NF6 to NF9 depicted in Figure 5 demonstrated the sustained action of the tablet in comparison to the core tablet. As per above discussion, the drug release from the batch NF6 provides release in small intestine due to lack of Eudragit S-100 coating. However, formulations NF7 and NF8 retarded the drug release with initial burst release with lag time of 8 h. The combination of Eudragit RSPO : RLPO coated tablet with Eudragit S-100 coating (7.5% w/v) provides sustained action and no burst release, but on the other hand, this formulation showed less cumulative release profile. This phenomenon may be attributed to the combination of good erodible properties of Eudragit S-100 and the swelling behaviour of RLPO and RSPO polymer [42, 43].

 The drug delivery systems targeted to the colon should not only protect the drug from being released in the physiological environment of stomach and small intestine but also release the drug in colon after enzymatic degradation by colonic bacteria. Hence, *in vitro* drug release studies were carried out for selected formulations in SIF (pH 7.4) containing 4% w/v of rat caecal contents. The rat caecal contents were included to mimic the colonic environment. At the end of 24 h of testing which includes testing in simulated gastric and intestinal fluids, the percent of drug released from naproxen tablets coated with coat formulation NF4 was found to be increase from 6 h onwards indicating the commencement of breaking of coats. The percent of drug released after 24 h of testing was 98.10 ± 1.61% which indicates that there was more drug released in the presence of enzymes as compared to dissolution without rat caecal content. Hence, it was concluded that the dissolution study in the presence of rat caecal content resulted in improved drug release because the physiology of rat colon is similar to that of the human colon. Therefore the higher amount of drug release was observed [44].

 The photomicrographs of coated tablets (Figure 7(a)) before the dissolution study showed the presence of non porous, compact, and homogenous structure due to the coating layer of both HPC and Eudragit S-100 polymer. After 2 h of dissolution of tablet in pH 1.2 media, it was observed that the tablets remain in intact form with a homogenous structure (Figure 7(b)) due to the presence of pH-dependent polymer which does not degrade in acidic media. However, when tablets come in contact with phosphate buffer pH 6.8, at the end of 3 hrs rare small tiny pores were observed (Figure 7(c)), indicating the formation of a gelling structure through which solvent starts to penetrate into the porous network and hence results in the formation of tiny pores. These pores further got increased in both number and diameter in phosphate buffer pH 7.4 followed by dissolution of coating layer (Figure 7(d)), allowing the hydrophilic membrane to be exposed to the solvent which further accounts for the rupturing of the membrane indicating that both diffusion and erosion mechanism are responsible for release of drug. Hence, the results obtained from SEM studies of tablets may support the *in vitro* drug release profile as mentioned in Figure 7 [11]. 

 The coating thickness is inversely proportional to the drug release. As the concentration of Eudragit S-100 coating increases, the drug release decreases [45]. This might be due to the fact that the more time taken by the coat to get erode when the coating concentration was increased. The erosion study suggests that 2.5% w/v Eudragit S-100 coating was insufficient to sustain the release more than 5 h due to less resistant to the erosion. However, this shows that when the enteric coating gets solubilized, the press-coated tablets get exposed to the media which release the drug at insufficient lag time. On increasing the coating concentration to 5% w/v, the coat was found to be less eroded after 12 h, giving the lag time of 6 h which is sufficient to delay the drug release to reach the colon. The drug released slowly from this system which may be attributed to its sustained action and may be due to diffusion mechanism. On further increasing coat concentration to 7.5% w/v, the lag time of 8 h was obtained which suggests that the erosion of coat occurs at a very slow rate due to high coating thickness. Taking this into consideration, it was revealed that the coating level of 5.0% w/v was found to be optimum, because at this concentration the required lag time with sustained action of the drug release was observed. 

Among the all the formulations, the formulations NF1, NF2, and NF6 followed Fickian kinetics (*n* ≤ 0.45), and the remaining formulations followed non-Fickian kinetics (*n* ≥ 0.45). This indicates that the release of naproxen sodium release from matrix tablets is diffusion for NF1, NF2 and NF6 formulations, whereas the release of naproxen sodium from remaining formulation follows the anomalous transport from matrix tablets. 

The stability studies concluded that the drug degradation follows first-order kinetics. The optimized formulation NF4 showed the shelf life of more than two years which indicates that there is no need to add overages to ensure 2-year shelf life. 

## 5. Conclusion

The prepared formulation was evaluated using various standard tests, and from the results it was concluded that the coating combination of both polymer, that is, HPC and Eudragit S-100, was successful in preventing the drug release in the upper part of gastrointestinal tract. The *in vitro* drug release studies and SEM analysis demonstrated that the optimized formulation proved to be a promising drug delivery for colon targeting. The release kinetics of all the batches were best fitted to Korsmeyer-Peppas model and Higuchi model. The stability data suggested the lowest degradation and maximum shelf life as per ICH guidelines.

## Figures and Tables

**Table 1:** Composition of naproxen sodium core, press-coated tablets, and enteric-coated tablets.

Ingredients	NF1	NF2	NF3	NF4	NF5	NF6	NF7	NF8	NF9
Drug	+	+	+	+	+	+	+	+	+
Lactose	+	+	+	+	+	+	+	+	+
Starch	+	+	+	+	+	+	+	+	+
Magnesium Stearate	+	+	+	+	+	+	+	+	+
HPC	−	+	+	+	+	−	−	−	−
RSPO : RLPO	−	−	−	−	−	+	+	+	+
*ED S-100 2.5% w/v	−	−	+	−	−	−	+	−	−
*ED S-100 5.0% w/v	−	−	−	+	−	−	−	+	−
*ED S-100 7.5% w/v	−	−	−	−	+	−	−	−	+

^+^Sign indicates presence of ingredient.

^−^Sign indicates absence of ingredient.

*Eudragit S-100.

**Table 2:** Micromeritic properties of granules of naproxen sodium core tablet.

Angle of repose (°) ± S.D	23.41 ± 1.32
Bulk density (gm/mL) ± S.D	0.36 ± 0.004
Tapped density (gm/mL) ± S.D	0.40 ± 0.003
Carr's index (%) ± S.D	9.84 ± 1.09
Hausner's ratio	1.15 ± 0.03

Mean ± S.D. (*n* = 3).

**Table 3:** Physicochemical parameters of naproxen sodium core, press-coated tablets, and enteric-coated tablets.

Formulation code	Weight variation (mg)	Thickness (mm)	Diameter (mm)	Hardness (kg/cm^2 ^)	Friability (%)	Drug content (%)
NF1	251.24 ± 2.24	4.01 ± 0.02	8.01 ± 0.02	4.09 ± 0.59	0.63 ± 0.03	95.27 ± 2.03
NF2	500.31 ± 2.26	4.62 ± 0.02	12.88 ± 0.02	6.48 ± 0.59	0.53 ± 0.02	92.36 ± 1.91
NF3	501.19 ± 2.19	4.78 ± 0.02	12.03 ± 0.02	6.05 ± 0.59	0.25 ± 0.03	96.37 ± 2.09
NF4	502.15 ± 2.21	4.82 ± 0.02	12.08 ± 0.02	6.54 ± 0.67	0.28 ± 0.02	93.99 ± 1.48
NF5	502.07 ± 2.26	4.86 ± 0.02	12.92 ± 0.02	6.47 ± 0.59	0.20 ± 0.02	95.61 ± 1.95
NF6	503.15 ± 2.23	4.57 ± 0.02	12.81 ± 0.02	6.34 ± 0.67	0.46 ± 0.02	94.49 ± 1.86
NF7	500.68 ± 2.28	4.53 ± 0.02	12.86 ± 0.02	6.25 ± 0.59	0.29 ± 0.02	94.41 ± 1.90
NF8	501.63 ± 2.28	4.57 ± 0.02	12.74 ± 0.02	6.59 ± 0.67	0.23 ± 0.02	95.61 ± 1.96
NF9	502.03 ± 2.26	4.59 ± 0.02	12.94 ± 0.02	6.35 ± 0.67	0.22 ± 0.01	94.36 ± 2.03

Mean ± S.D. (*n* = 3).

**Table 4:** Regression coefficient (*r* ^2^) values of drug release data calculated from various drug release kinetic models and *n* value in accordance with Korsemeyer-Peppas.

Formulation code	Zero order	First order	Higuchi	Korsemeyer-Peppas	
	*r* ^2^	*r* ^2^	*r* ^2^	*r* ^2^	*n*
NF1	0.892	0.986	0.990	0.979	0.399
NF2	0.896	0.986	0.992	0.976	0.419
NF3	0.950	0.884	0.988	0.978	0.847
NF4	0.916	0.976	0.993	0.970	0.786
NF5	0.951	0.956	0.987	0.984	0.823
NF6	0.919	0.977	0.980	0.975	0.401
NF7	0.934	0.957	0.959	0.951	0.693
NF8	0.949	0.930	0.958	0.949	0.728
NF9	0.954	0.955	0.956	0.953	0.735
